# Methods and guidance on conducting, reporting, publishing, and appraising living systematic reviews: a scoping review

**DOI:** 10.1186/s13643-023-02396-x

**Published:** 2023-12-14

**Authors:** Claire Iannizzi, Elie A. Akl, Eva Anslinger, Stephanie Weibel, Lara A. Kahale, Abina Mosunmola Aminat, Vanessa Piechotta, Nicole Skoetz

**Affiliations:** 1 Institute of Population Health, Faculty of Medicine and University Hospital Cologne, University of Cologne, Cologne, Germany; 2 Department of Medicine, American University of Beirut, Beirut, Lebanon; 3 Department of Health Research Methods, Evidence, and Impact, McMaster University, Hamilton, Canada; 4 Evidence-Based Medicine, Department I of Internal Medicine, Center for Integrated Oncology Aachen Bonn Cologne Duesseldorf, Faculty of Medicine, University Hospital Cologne, University of Cologne, Kerpener Str. 62, 50937 Cologne, Germany; 5 Department of Anaesthesiology, Intensive Care, Emergency and Pain Medicine, University Hospital Würzburg, Würzburg, Germany; 6 Editorial and Methods Department, Cochrane Central Executive, Cochrane, St Albans House, 57-59 Haymarket, London, SW1Y 4QX UK; 7 Rafic Hariri School of Nursing, American University of Beirut, Riad El Solh, P.O. Box 11-0236, Beirut, 1107 2020 Lebanon

**Keywords:** Living systematic reviews, Methods and guidance, Scoping review, Conducting LSRs, Reporting, Appraisal

## Abstract

**Background and objective:**

The living systematic review (LSR) approach is based on ongoing surveillance of the literature and continual updating. Most currently available guidance documents address the conduct, reporting, publishing, and appraisal of systematic reviews (SRs), but are not suitable for LSRs per se and miss additional LSR-specific considerations. In this scoping review, we aim to systematically collate methodological guidance literature on how to conduct, report, publish, and appraise the quality of LSRs and identify current gaps in guidance.

**Methods:**

A standard scoping review methodology was used. We searched MEDLINE (Ovid), EMBASE (Ovid), and The Cochrane Library on August 28, 2021. As for searching gray literature, we looked for existing guidelines and handbooks on LSRs from organizations that conduct evidence syntheses. The screening was conducted by two authors independently in Rayyan, and data extraction was done in duplicate using a pilot-tested data extraction form in Excel. Data was extracted according to four pre-defined categories for (i) conducting, (ii) reporting, (iii) publishing, and (iv) appraising LSRs. We mapped the findings by visualizing overview tables created in Microsoft Word.

**Results:**

Of the 21 included papers, methodological guidance was found in 17 papers for conducting, in six papers for reporting, in 15 papers for publishing, and in two papers for appraising LSRs. Some of the identified key items for (i) conducting LSRs were identifying the rationale, screening tools, or re-revaluating inclusion criteria. Identified items of (ii) the original PRISMA checklist included reporting the registration and protocol, title, or synthesis methods. For (iii) publishing, there was guidance available on publication type and frequency or update trigger, and for (iv) appraising, guidance on the appropriate use of bias assessment or reporting funding of included studies was found. Our search revealed major evidence gaps, particularly for guidance on certain PRISMA items such as reporting results, discussion, support and funding, and availability of data and material of a LSR.

**Conclusion:**

Important evidence gaps were identified for guidance on how to report in LSRs and appraise their quality. Our findings were applied to inform and prepare a PRISMA 2020 extension for LSR.

**Supplementary Information:**

The online version contains supplementary material available at 10.1186/s13643-023-02396-x.

## Introduction

Systematic reviews (SRs) are essential to provide evidence-based answers to clinical and public health-related questions. Due to the continuous publishing of relevant primary studies in some areas, it is important to keep these SRs up-to-date [1]. One could achieve that goal by adopting the living systematic review (LSR) approach, which is based on an ongoing surveillance of the literature and continual updating [2]. Regular searches ensure that the SR includes the latest available evidence and remains up-to-date [2]. Therefore, LSRs are most suitable for high-priority topics with substantial uncertainty and frequent publications. When continually updating a review, it is important to report changes to the methodology and the findings in transparent and traceable ways, which can be challenging.

Few guidance documents address the conduct, reporting, publishing, and appraisal of LSRs. The Living Evidence Network developed in 2019 the “Guidance for the production and publication of Cochrane living systematic reviews” [3]. However, this guidance lacks certain aspects of the LSR methodology, which have been shown to be important in the last years with the rising number of LSRs conducted. While the recent update of the “Preferred Reporting Items for Systematic reviews and Meta-Analyses” (PRISMA) can be used for reporting LSRs, the statement indicates there may be some additional considerations that need to be addressed [4]. Also, the AMSTAR 2—Assessing the Methodological Quality of Systematic Reviews—tool [5] which was developed for the critical appraisal of the quality of SRs, does not consider LSRs.

Therefore, it is of high interest to summarize the literature evaluating methods of conducting, reporting, publishing, and appraising LSRs, as well as any guidance on those methods. Scoping reviews are particularly useful in the context of emerging evidence and act as a precursor for other topic-related projects [6]. This scoping review is part of a larger project to develop an extension of the PRISMA 2020 statement for living systematic reviews.

## Objective

The main objective is to systematically collate methodological literature on guidance on how to conduct, report, publish, and appraise the quality of LSRs and to systematically map how much and what kind of evidence is currently available.

## Methods

A protocol elaborating on the detailed methodology of this scoping review was already published [7]. The main differences in methods between the protocol and this scoping review are displayed in the Supplementary Table 1.

### Scoping review methodology

To achieve the objective, we conducted a scoping review to identify and evaluate existing evidence and map the availability of methods papers, evidence gaps, and associated primary research gaps [6]. We followed the standard scoping review methodology guidance of the Joanna Briggs Institute [6] and applied the following steps:

- Identification of the research question
- Identification of relevant studies
- Study selection
- Charting the data
- Collating, summarizing, and reporting of the results [8]

Moreover, we adhered to the Preferred Reporting Items for Systematic Reviews and Meta-Analyses extension for Scoping Reviews (PRISMA-ScR) checklist (see Supplement Table 2) for transparent reporting of the results [9].

### Eligibility criteria

We included articles that devoted at least one paragraph to discuss methods or conceptual approaches on how to conduct, report, publish, or appraise LSRs. Such articles were ideally methodological or concept papers describing methods for LSRs, guidance (e.g., handbooks) for undertaking LSRs, issued by organizations that conduct evidence syntheses, and commentaries or editorials that discuss methods for LSR.

We excluded from our search, LSRs themselves, LSR protocols, and non-LSR-specific papers.

### Identification of relevant studies

We searched MEDLINE (Ovid), EMBASE (Ovid), and The Cochrane Library. All searches were completed on August 28, 2021, and we searched from database inception. The search strategy was initially developed by a researcher experienced in developing literature search strategies with support from an information specialist (LH), as part of a larger project to develop an extension of the PRISMA 2020 statement for LSRs [10, 11]. The strategy was peer-reviewed and updated by another information specialist (IM). Please see Box 1 of the Appendix for the complete search strategy.

As for searching the “gray literature,” we looked for existing guidelines and handbooks on LSRs from organizations that conduct evidence syntheses (e.g., Cochrane handbook, Living Evidence network, JBI) using the Lens.org website. Additionally, we conducted an ancestry search to identify relevant LSR handbooks and guidance documents from the reference list of published LSRs. We performed a descendency search, using certain seminal documents (e.g., papers defining LSRs and Cochrane guidance), and tracked their citations via Google Scholar.

### Article selection

Two authors (from among CI, NS, EA) contributed to screening independently and in duplicate titles and abstracts. We used a web-based systematic review software Rayyan (RRID:SCR_017584) for the screening process. To ensure a consistent screening procedure and optimize agreement, we developed and used a detailed written instruction form. We then screened for full text assessing eligibility, based on our predefined eligibility criteria. Disagreements and conflicts were solved by consulting a third author.

### Data extraction and presentation

Two review authors (from among CI, NS, VP, SW, EA) extracted and cataloged the data on LSR-specific methodological aspects into a standardized and pilot-tested data extraction form in Microsoft Excel (RRID:SCR_016137). We extracted the main article characteristics and LSR-specific guidance data according to our predefined categories on (i) conducting, (ii) reporting, (iii) publishing, and (iv) appraising LSRs. The identified evidence was mapped by visualizing overview tables created in Microsoft Word. The items of the conducting category are based on the standard process of conducting a systematic review from the Cochrane Handbook [12], including the intermediate steps from describing the rationale to evidence synthesis. The reporting category includes the 27 items of the original PRISMA 2020 checklist [4] to identify whether LSR-specific reporting guidance exists for each of these items. The items of the publishing category are partly based on standard Cochrane guidance for systematic reviews [12] and the experiences of LSR authors within this author team. The LSR appraisal category is based on the 16 questions from the AMSTAR 2 tool [5]. Even though we extracted and classified the data according to these categories, we considered that items from one category (e.g., conducting LSR) could have an impact on items from another category (e.g., publishing LSR) and might even overlap. The extracted study characteristics and category items are listed in Supplementary Table 3.

## Results

**Fig. 1:**
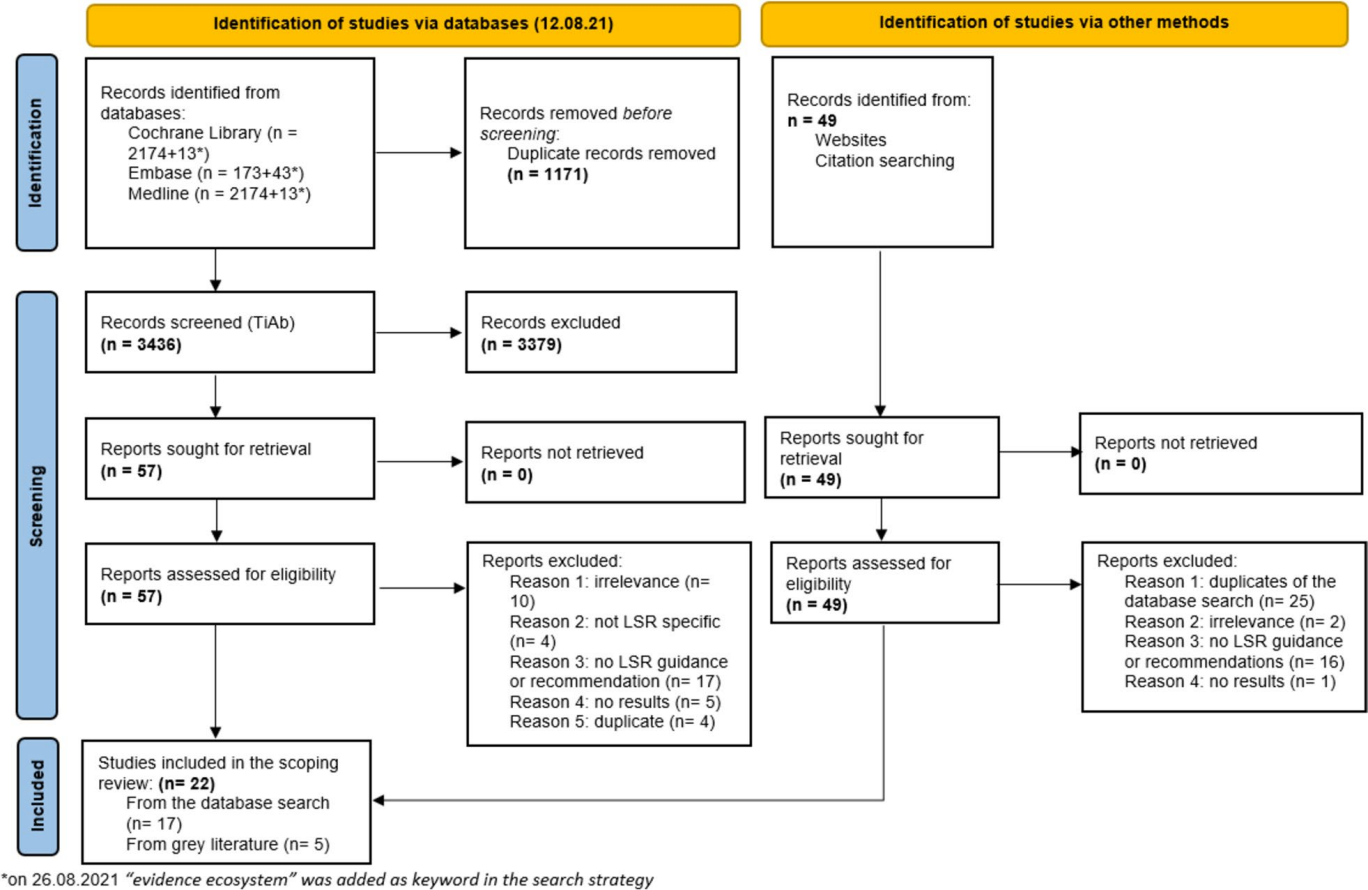
Flowchart of the database search and gray literature

We identified 4590 references, potentially relevant to our research question. After having removed 1171 duplicates, we screened 3436 records on title and abstract and excluded 3379 records that did not meet the pre-defined eligibility criteria. We screened the full text of the remaining 57 records and included 17 papers from the database search in the scoping review. We also searched for “gray literature” and identified 49 potential records, from which we included five papers in the scoping review. In total, 21 articles from both searches were included in the scoping review. The detailed selection process and results are reported in the PRISMA flow diagram (see Fig. 1) [4].

### The evidence map

The 21 included papers provided data for 40 of our pre-defined LSR-specific items. Methodological guidance was found in 17 papers for conducting LSRs, in six papers for reporting LSRs, in 15 papers for publishing LSRs, and in two papers for appraising LSRs (see Tables 1 and 2).

**Table 1:** Evidence map on the four categories of LSR guidance

Papers	Extracted data: aim and guidance evidence				
	**Paper-specific aims**	**Conducting LSRs**	**Reporting LSRs**	**Publishing LSRs**	**Appraising LSRs**
**Brooker 2019 **[3] Guidance for the production and publication of Cochrane living systematic reviews: Cochrane Reviews in living mode	To provide detailed guidance on the production and publication process of a Cochrane living systematic review	√	√	√	√
**Crequit 2020 **[13] Future of evidence ecosystem series: 2. current opportunities and need for better tools and methods	To consider how the access to new sources and data types and the recent developments of new methods, technologies, and tools presents a great opportunity to create and sustain an ecosystem designed to support the production of updated high-quality evidence syntheses	√		√	
**Elliott 2014 **[1] Living Systematic Reviews: An Emerging Opportunity to Narrow the Evidence-Practice Gap	To describe several recent developments that have the potential to improve dramatically the efficiency of conventional SRs and enable the widespread production of LSRs			√	
**Elliott 2017 **[14] Living systematic review: 1. Introduction—the why, what, when, and how	To introduce what LSRs are and discuss the main issues in LSRs, including searching, updating scenarios, production processes, editorial and peer review, and publication	√		√	
**Harrington 2021 **[15] COVID-19 Technology-Enabled Living Systematic Reviews to Enhance Knowledge Translation	To provide details on how machine learning can be used for LSR and nursing implications	√		√	
**Kahale 2021 **[11] Tailored PRISMA 2020 flow diagrams for living systematic reviews: a methodological survey and a proposal	To assess how published LSRs report on the flow of studies through the different phases of the review for the different updates and to propose an approach for documentation and reporting		√		
**Lansky 2020 **[16] Living Systematic Reviews and Other Approaches for Updating Evidence	To introduce a new method for updating SRs called “living” SRs and indications for updating	√		√	
**Lerner 2019 **[17] Automatic screening using word embeddings achieved high sensitivity and workload reduction for updating living network meta-analyses	To develop and evaluate an algorithm for automatically screening citations when updating living network meta-analysis (NMA)	√			
**MacDonald 2020 **[18] Living systematic reviews at The BMJ	To give insights into how living SRs (in fast-moving research areas) at The BMJ will be handled by the research team and of their usual methodological standards	√			
**Millard 2019 **[19] Feasibility and acceptability of living systematic reviews: results from a mixed-methods evaluation	To provide details on the feasibility of LSRs, barriers, and facilitators	√		√	
**Negrini 2021 **[20] A systematic review that is “rapid” and “living”: A specific answer to the COVID-19 pandemic	To describe “rapid living” systematic reviews, an innovative methodological design used to systematically synthesize emerging evidence in the field of rehabilitation during the COVID-19 pandemic	√		√	
**Page 2020 **[4] The PRISMA 2020 statement: An updated guideline for reporting systematic reviews	To provide an updated guideline for reporting SRs with the PRISMA 2020 checklist and statement		√		
**Ravaud 2020 **[21] Future of evidence ecosystem series: 3. From an evidence synthesis ecosystem to an evidence ecosystem	To introduce a new approach and innovative solution to the current problems of need to provide up-to-date evidence synthesis for a specific clinical question	√	√	√	
**Simmonds 2017 **[22] Living systematic reviews: 3. Statistical methods for updating meta-analyze	To compare and consider the application of four methods to avoid specific statistical problems when updating meta-analyses for LSRs	√		√	
**Slaugther 2015 **[23] Enabling Living Systematic Reviews and Clinical Guidelines through Semantic Technologies	To provide a brief review of various efforts to produce semantic technologies for sharing and reusing content from clinical investigations (RCTs and other clinical primary studies)			√	
**Ter Schure 2019 **[24] Accumulation bias in meta-analysis: the need to consider time in error control	To investigate various ways in which time influences error control in meta-analysis testing and to introduce an Accumulation Bias Framework	√			√
**Thomas 2017 **[25] Living systematic reviews: 2. Combining human and machine effort	To specifically focus on ways in which the use of new human and machine “technologies” can make the standard SR process more efficient	√			
**Thomas 2021 **[26] Cochrane Handbook Chapter 22: Prospective approaches to accumulating evidence	To provide detailed guidance on prospective approaches to accumulating evidence for LSRs	√		√	
**Vergara-Merino 2020 **[27] Living systematic review: new inputs and challenges	To describe LSR’s relevance, the considerations that should be taken when producing one, and the challenges proper of this type of review	√	√	√	
**Winters 2020 **[28] Stay alive! What are living systematic reviews and what are their advantages and challenges?	To introduce living SRs and to discuss its advantages and challenges	√		√	
**Xu 2020 **[29] A brief INTRODUCTION to living systematic reviews (Chinese)	To introduce the development, characteristics, conditions, implementation, and applications of living systematic reviews	√	√	√	
**Total number**		**17/21**	**6/21**	**15/21**	**2/21**

**Table 2:** Summary of the number of papers reporting on each category, reported pre-defined items/sub-items, and gaps of guidance evidence

	**Conducting LSRs**	**Reporting LSRs**^**a**^	**Publishing LSRs**	**Appraising LSRs**^**b**^
N of papers reporting on a category	17 of 21	6 of 21	15 of 21	2 of 21
N of pre-defined items/sub-items reported on	19 of 21 sub-items	13 of the 27 PRISMA items	14 of 14 sub-items	4 of 18 (16 AMSTAR two tool questions & 2 additional items)
Pre-defined items/sub-items reported by at least half of identified papers	LSR rationale, Screening tools	Registration & protocol	Publication type, publication frequency, update publication trigger and time point for transitioning out of the living mode	Use of appropriate risk of bias assessment technique and funding of included studies reported
Pre-defined items/sub-items reported by less than half of identified papers	Changing of inclusion criteria, search (frequency, database and who), data extraction (frequency, who and how), quality and bias assessment (frequency and how), data synthesis with meta-analysis if applicable (frequency, who and how), frequency of the certainty of evidence assessment, authorship changes, ongoing method support and funding	PRISMA items on reporting: title (1), abstract (2), rationale (3), objective (4), eligibility criteria (5), information sources (6), search strategy (7), selection process (8), bias assessment (11), synthesis methods (13), study selection (16) and competing interests (26)	Type of information in an update, publication of review status, new citation & added to PubMed, publication of an update, specific time point of publication, when starting living mode publication, publication of between updates information, transition out of living mode trigger, peer review updates, publish authorship changes, publish PRISMA flow diagram	Assessment of protocol and review differences, use of comprehensive search strategy, and appropriate methods for meta-analysis
Identified gaps of guidance evidence	Who carries out bias assessment & certainty of evidence assessment	PRISMA items on reporting: data collection process (9), data items (10), effect measure (12), reporting bias assessment (14), certainty assessment (15), reporting of results, including study characteristics (17), presenting risk of bias in studies (18), results of individual studies (19), results of synthesis (20), reporting bias (21) and certainty of evidence (22), discussion (23a, 23bc and 23d) and reporting support and funding (25) the availability of data and material (27)	None	Eligibility criteria, explain study selection, assessments in duplicate, list of studies, funding, heterogeneity, RoB impact, preprints

^a^For the reporting category, the pre-defined items and sub-items were based on the existing PRISMA 2020 checklist (Page, et al. 2021)

^b^For the appraisal category, the pre-defined items were based on the AMSTAR 2 tool questions (SHEA, et al. 2017)

### LSR conducting guidance

From the 17 papers including guidance on conducting LSRs, we mapped and summarized the reported guidance for each of our pre-defined items and sub-items (see Table 3). We found evidence for all the pre-defined items on conducting and almost all the sub-items. A particular high frequency of papers, more than half of the 17 included papers, provided guidance on certain sub-items such as the rationale for conducting a LSR and the screening tool of the search. Between one and five papers presented guidance on other sub-items, including changing and re-evaluating the inclusion criteria, the search (frequency, database, and who), the data extraction (frequency, who, and how), the quality and bias assessment (frequency and how), the data synthesis with meta-analysis if applicable (frequency, who, and how), the frequency of the certainty of evidence assessment, authorship changes, ongoing method support, and funding. Also, we found that some papers established very broad guidance on several steps of conducting a LSR [1, 3, 13–15, 25, 29]. The remaining papers reported more specific guidance on certain particular steps of the LSR conduction process. We could not identify any evidence for guidance on two sub-items: who carries out the quality and bias assessment and the certainty of evidence assessment.

**Table 3:** Evidence table on identified guidance for conducting LSRs with a narrative summary of extracted data

Items of guidance	Subgroups of items & *N of* papers	Narrative summary of extracted data
**Criteria/rationale for conducting LSR**	Rationale (*N* = 10/17)	• High prevalence of condition/RQ [13, 15] • Existing results change [3, 15] • Priority for decision making [3, 20, 22, 26, 27, 29] • Low certainty of evidence or rapidly emerging evidence [3, 15, 18, 21, 26, 27, 29]
**Inclusion criteria**	Emerging change (*N* = 1/17)	• Adaption is needed, if inclusion criteria are changed [3]
	Re-evaluate (*N* = 2/17)	• Based on the evolving quality of evidence, a new understanding of context, with the involvement of experts with different expertise [20] • Identify and re-define most relevant RQs [13]
**Search**	Frequency (*N* = 8/17)	• Set up auto alerts to provide a regular feed of new citations [14] • Continuous search (e.g., varying between weekly and monthly) [1, 3, 13, 14, 16, 19, 28, 29]
	Database (*N* = 2/17)	• Bibliographic databases, clinical trials registries, gray literature [3, 14]
	Who (*N* = 1/17)	• Information specialists or librarians, using technological enablers [3]
	Screening tool (*N* = 10/17)	• Computer-supported & automated [3, 13–15, 17, 19, 26–29] • Continuous database search with push notification [25, 26] • Guidance on eligibility: machine-learning classifier, crowdsourced inclusion decisions [25]
**Data extraction**	Frequency (*N* = 3/17)	• Continuous search (trigger-dependent) [1] • Immediately after study identification [22] • Once new evidence has been identified for inclusion, the update process including data extraction starts [29]
	Who (*N* = 1/17)	• Machine-learning information-extraction systems [25] • Linkage of existing structured data sources (e.g., clinical trials registries) [25]
	How (*N* = 6/17)	• AI, machine learning, and automated structured data [3, 13, 15, 26, 29] • Crowd-sourcing [13, 26, 27]
**Quality & bias assessment**	Frequency (*N* = 2/17)	• Regular updating, at a defined time interval [3] • Once new evidence has been identified for inclusion, the update process including RoB assessment starts [29]
	Who (*N* = 0/17)^a^	
	How (*N* = 2/17)	• Machine learning-assisted RoB tools (e.g., RobotReviewer) [25] • AI-assited tools [26]
**Data synthesis with meta-analysis (if applicable)**	Frequency (*N* = 5/17)	• Immediately after new study inclusion [22, 24] • When deciding to update [14], on a continuous base [1] • Once new evidence has been identified for inclusion, the update process including data synthesis starts [29]
	Who (*N* = 1/17)	• People responsible for performing the initial evidence synthesis [21]
	How (*N* = 5/17)	• AI, e.g., automatic text generation tools [3] • Error controls, e.g., by trial sequential analysis [24, 29], sequential methods, or Bayesian framework [1] • Follow the description of the planned statistical approach to update a meta-analyze [14]
**Certainty of the evidence assessment**	Frequency (*N* = 1/17)	• Regular updating [3]
	Who (*N* = 0/17)^a^	
**Authorship changes**	Authorship (*N* = 4/17)	• Regularly updated for each new review version, according to contribution [1, 3] • Contribution of each member of the group was assessed as sufficient for authorship (and meeting ICMJE criteria) or not [14, 29]
**Ongoing method support**	Method support (*N* = 2/17)	• Involvement of different methodological expertise [20] • Team of clinicians, researchers, and graduate students with SR expertise [29]
**Funding**	Funding (*N* = 4/17)	• Impact on maintaining LSR [3] • Direct funding for personnel [19], a consistent flow of funding to research groups is needed [13, 16]

^a^The two items for which no data could be identified are grayed out

### LSR reporting guidance

From the six papers providing guidance on reporting LSRs, we mapped the available data for each of the PRISMA items and sub-items and summarized the identified guidance (see Table 4). We found guidance on 13 out of the 27 PRISMA items for reporting a LSR. We identified a higher frequency of papers, three out of the six, providing guidance for PRISMA item 24 on the registration and protocol. One or two papers provided guidance for PRISMA items one until eight, 11, 13, 16, and 25. We noted that one paper [3] included particularly elaborated guidance on some of the PRISMA items, and the remaining papers provided guidance on a particular PRISMA item.

**Table 4:** Evidence table on identified guidance for reporting LSRs with narrative summary of extracted data

PRISMA items (number)	Subgroups of items & *N* of papers reporting evidence	Narrative summary of extracted data
**(1)Title**	*N* = 2/6	• Transition to and out of living mode must be recognized in the title, responsible parties must be informed [3] • Additional information regarding the indication of an update or “living” SR approach must be provided in the title [4]
**(2)Abstract**	*N* = 1/6	• Abstract must indicate identification as an LSR; updated search results must be reported [3]
**Introduction**	(3) Rationale (*N* = 1/6)	• Rational for the LSR approach: Previous updates must be mentioned [3]
	(4) Objective (*N* = 1/6)	• Previously performed updates must be mentioned [3]
**Methods**	(5) Eligibility criteria (*N* = 1/6)	• Remain the same as for standard SRs [3]
	(6) Information sources (*N* = 1/6)	• Accurate reporting is necessary, including the PRISMA flow diagram [3]
	(7) Search strategy (*N* = 1/6)	• Must be specified and reported in the protocol [3]
	(8) Selection process (*N* = 1/6)	• Report whether any new citations retrieved by the monthly searches was immediately screened; using technical support tools [3]
	(9) Data collection process (*N* = 0/6)^a^	
	(10) Data items (*N* = 0/6)^a^	
	(11) Study risk of bias assessment (*N* = 1/6)	• Report the use of machine learning and automated structured data extraction tools [3]
	(12) Effect measure (*N* = 0/6)^a^	
	(13) Synthesis methods (*N* = 2/6)	• Specify statistical methods used to correct type 1 and 2 errors [27] • Enables for data synthesis [3]
	(14) Reporting bias assessment (*N* = 0/6)^a^	
	(15) Certainty assessment (*N* = 0/6)^a^	
**Results**	(16) Study selection (*N* = 1/6)	• Record in detail the search results, a spreadsheet is recommended. Present either the results of the base and updates separately, all combined or only the updated versions combined [11]
	(17) Study characteristics (*N* = 0/6)^a^	
	(18) Present risk of bias in studies (*N* = 0/6)^a^	
	(19) Present results of individual studies (*N* = 0/6)^a^	
	(20) Results of synthesis (*N* = 0/6)^a^	
	(21) Reporting bias (*N* = 0/6)^a^	
	(22) Certainty of evidence (*N* = 0/6)^a^	
**Discussion (23)**	(23a) General interpretation (*N* = 0/6)^a^	
	(23bc) Limitations (*N* = 0/6)^a^	
	(23d) Implications for practice (*N* = 0/6)^a^	
**Registration and protocol (24)**	*N* = 3/6	• Justify the use of the “living” format in their protocol and mention pre-established criteria to abandon the “living” format for the conventional method [27] • Based on SR protocol [29] and the use of a template on how to create protocol [3]
**Support and funding (25)**	*N* = 0/6^a^	
**Competing interests (26)**	*N* = 1/6	• Role of each work group member and their COI should be transparent [21]
**Availability of data & material (27)**	*N* = 0/6^a^	

^a^Items for which no evidence was identified are grayed out

We could not identify any guidance for the PRISMA items on reporting the methods, including data collection process (9), data items (10), effect measure (12), reporting bias assessment (14), and certainty assessment (15). Further, there was no guidance identified for the reporting of results, including study characteristics (17), presenting the risk of bias in studies (18), results of individual studies (19), results of synthesis (20), reporting bias (21), and certainty of evidence (22). No data was found on reporting the three items (23a, 23bc, and 23d) of the discussion, on the item reporting support and funding (25), and on the availability of data and material (27).

### LSR publishing guidance

From the 15 papers including guidance on publishing LSRs, we mapped the available data for our pre-defined items and sub-items and summarized the identified guidance (see Table 5). We found guidance for all of the pre-defined items and all the sub-items. We identified a particular high frequency of papers, more than half of the 15 included papers, providing guidance on certain sub-items such as the publication type, publication frequency, update publication trigger, and time point for transitioning out of the living mode. A lower frequency of papers included guidance on the remaining sub-items. Also, we note that some papers provide very broad guidance on several aspects of publishing a LSR [3, 14, 19, 29]. The other remaining papers provided more specific guidance on particular steps of the LSR publication process.

**Table 5:** Evidence table on identified guidance for publishing LSRs with a narrative summary of extracted data

Items of guidance	Subgroups of items & *N* of papers reporting evidence	Narrative summary of extracted data
**Publication type of new findings**	Publication types *N* = 9/15	• Latest findings published on website [14, 28] • Depending on changes for the conclusion (major changes: new DOI and citation) [1] • Interactive living evidence map and dynamic table [20] • What’s a new table, update alert [3] • Full review update [19] • [21, 23, 29]]
	Type of information in an update *N* = 2/15	• The format of LSR publication and dissemination must accommodate its frequent updates [29] • Date of last search, numbers of citations screened, studies awaiting inclusion [14]
**Publication of review status**	*N* = 4/15	• Regular and transparent statements [3], alerts [14] • Monthly/daily/three monthly statements to reader about review status [3, 19] • Status and information of the update process should be disclosed to users, and the update results should be published in a timely manner [29]
**New citation & added to PubMed**	*N* = 5/15	• DOI & citation adaption as appropriate [3, 19] • Depending on changes for the conclusion (major changes: new doi and citation) [1, 27] • [29]
**Publication of an update**	Publication frequency *N* = 8/15	• Regular updating process [3] • Trigger dependent [13, 14, 19, 22, 26, 29] • When a certain number of new publications [28]
	Specific time point of publication *N* = 5/15	• Between immediately when new evidence is identified to every 4 or 6 months [3, 14, 19, 29] • Explicit and a priori commitment to a predetermined frequency of review updating [22]
	Updating trigger *N* = 7/15	• Criteria-dependent (evidence dependent) [28] • When new information is likely to impact the review conclusion [3, 14, 26] • Independent from trigger, when new evidence is identified [19, 22, 29]
	When starting living mode publication *N* = 4/15	• Priority & relevance dependent [3, 19] • Happens when the normal SR is released or this action usually occurs when the normal SR is released or updated [29] • (1) new priority of topic, (2) inadequate evidence available, and (3) research moving quickly and emerging evidence impacting conclusion [15]
**Publication of between updates information**	*N* = 4/15	• Interactive living evidence map and dynamic table [20] • When new evidence is included: the reader should be notified of the process [3, 14, 19]
**Transition out of living mode**	Time point *N* = 7/15	• Evidence/trigger dependent [3, 29] • Specific thresholds for transitioning out of a Living systematic review mode, if known. [14] • When “enough evidence” but statistically unreasonable anymore [13, 16, 22] • Explicit discouraged from editor/journal [21]
	Transition out trigger *N* = 6/15	• When no rapidly iterating and new evidence is emerging, no priority [21, 29] • Evidence unlikely to change conclusion [3, 13, 16, 22]
**Peer review updates**	*N* = 5/15	• Peer review [3], dependent on update [14, 19] • Depending on whether new studies are identified and if new studies are included, then evidence impacts on conclusion [1] • Inclusion of new evidence requires editorial and optional peer review [29]
**Publish authorship changes**	*N* = 4/15	• LSR publication should have an appropriate author labeling mechanism, and all authors should conform to the ICMJE specification [14, 29] • Transparent and appropriate contribution fulfilling authorship criteria [1, 3]
**Publication of Prisma flow diagram**	*N* = 2/15	• Should regularly be updated [3], evidence-dependent to see live progress [19]

### LSR appraisal guidance

From the two papers including guidance on LSRs appraisal, we mapped the available data for each AMSTAR 2 tool question and some additional items and summarized the identified guidance (see Table 6). We found guidance on appraising LSRs for four of the pre-defined items. Among the two included papers, both provided guidance on the use of appropriate risk of bias assessment techniques and funding of included studies reported. One of each provided guidance on the assessment of protocol and review differences, the ongoing search, searched study registries, and gray literature. Moreover, we noted that one paper included more elaborated guidance on several aspects of quality appraisal [3]. We found no data for guidance on the remaining 14 items and two sub-items.

**Table 6:** Evidence table on identified guidance for appraising LSRs with a narrative summary of extracted data

Items of guidance & *N* of papers	Sub-items & narrative summary of extracted data
**(1)RQ & inclusion criteria** (*N* = 0/2)^a^	
**(2)Methods established prior to the conduct & justify deviation from the protocol** (*N* = 1/2)	Whether/how the difference between protocol and review was assessed: • Authors should note that the updated review includes additional methods pertaining to the LSR and refer the reader to the living systematic review protocol appendix [3]
	Whether/how the difference between review versions was assessed: no evidence^a^
**(3)Explain study selection** (*N* = 0/2)^a^	
**(4)Use of comprehensive search strategy** (*N* = 1/2)	• Ongoing search is recommended [3]
	Conducted search within a certain month of LSR completion: no evidence^a^
	• Searched study registries are recommended [3]
	• Searched reference list/gray literature is recommended [3]
**(5)Study selection in duplicate** (*N* = 0/2)^a^	
**(6)Data extraction in duplicate** (*N* = 0/2)^a^	
**(7)List of excluded studies & justification** (*N* = 0/2)^a^	
**(8)Adequate description of included studies** (*N* = 0/2)^a^	
**(9)Use of appropriate RoB assessment technique** (*N* = 2/2)	• Accumulation bias [24] • If new relevant methods emerged that would be appropriate to integrate into the methods it is recommended (risk of bias tools) new evidence will be assessed with risk of bias tool [3]
**(10)Funding of included studies reported** (*N* = 0/2)^a^	
**(11)Use of appropriate methods for meta-analysis** (*N* = 1/2)	• Refer to overview of the Framework for Adaptive Meta-analysis [3]
**(12)(if meta-analysis) assessment of potential RoB impact on pooled results** (*N* = 0/2)^a^	
**(13)Accounted for RoB when interpreting/discussing the results** (*N* = 0/2)^a^	
**(14)Explanation & discussion of heterogeneity observed in results** (*N* = 0/2)^a^	
**(15)(if quantitative synthesis) adequate investigation of publication bias & impact on result** (*N* = 0/2)^a^	
**(16)Report of potential COI sources (funding)** (*N* = 0/2)^a^	
**Use & handling of preprints** (*N* = 0/2)^a^	
**Guidance on using a specific checklist** (*N* = 0/2)^a^	

^a^Items for which no guidance was be identified are grayed out

## Discussion

To summarize the results, we included 21 articles from both search approaches in the scoping review. These papers included data for 40 of our pre-defined LSR-specific sub-items. Methodological guidance was found in 17 papers for conducting LSRs, in six papers for reporting LSRs, in 15 papers for publishing LSRs, and in two papers for appraising LSRs. We identified guidance on conducting LSRs for all of our pre-defined items of interest. Lacking evidence only exists for two sub-items on who carries out the quality and bias assessment and on the certainty of evidence assessment. Thus, we can state from our findings that there is enough guidance available in the literature on how to conduct a LSR and no major evidence gaps have been found.

We identified major evidence gaps in literature on guidance for reporting LSRs. There is lacking guidance for many of the PRISMA sub-items, such as reporting on the methods, the results, the discussion, reporting support and funding, and the availability of data and material. We did not find any evidence gaps in the literature for guidance on publishing LSRs. The identified papers included guidance for all of the pre-defined items and sub-items on publishing LSRs.

Regarding the literature on guidance for appraising the quality of LSRs, we can state that most of the important key items are lacking, indicating major evidence gaps. These include appraisal aspects on eligibility criteria, explaining study selection, assessments of data in duplicate, the list and description of included studies, funding sources and COI declarations reporting, assessing the heterogeneity of results, impact of risk of bias assessment on results, and use as well as handling of preprints.

This scoping review has certain limitations. The search was conducted in 2021 and within this 2-year gap, we could have failed to identify additional literature published since. We only focused on our four predefined categories of LSR methodological aspects, including conducting, reporting, publishing, and appraisal of LSRs. Even though these categories were drafted based on existing LSR methods handbooks, the PRISMA reporting checklist for SRs and the AMSTAR 2 tool for appraisal, a different author team may have chosen different categories or emphasized other LSR aspects. Moreover, we included quantitative guidance literature, rather than qualitative reviews or reports, as these would have sat outside the scope of this paper. The methodology of a scoping review itself includes some limitations as well. The scoping review is an approach to inform research and decision-making on existing evidence gaps and the availability of literature within a certain field of interest. The main purpose is to map, identify, and inform for future systematic reviews or other types of syntheses. Thus, the scope of a scoping review is often limited to presenting what kind of evidence exists, without further investigating and synthesizing the data of each included reference.

For the specific objective of our project, the scoping review approach has important strengths. We used a sensitive search strategy developed by an experienced researcher and information specialist. The article selection process, including the screening and data extraction that have been conducted independently and in duplicate, adds to the quality of the systematic approach. Also, the data extraction form was piloted before by the author group. We developed and published a detailed a priori protocol for this scoping review, which pre-defines our objective, the methods used, and the reporting of the review.

Our findings are of utmost importance, as they reveal important evidence gaps in methodological guidance on the reporting and quality appraisal of LSRs. We cannot provide any rational explanation as to why there is a lack of guidance for certain LSR-specific aspects, such as reporting and appraisal, and for other aspects, higher frequencies of guidance exist. We believe that the first obvious methodological question that authors need to address when a LSR becomes a relevant approach for their investigation, is how to conduct this novel review type. Thus, the need for LSR-specific guidance on conduct was probably acknowledged very early and researchers addressed this question in handbooks and guidance papers. Regarding the aspect of reporting or appraisal, guidance already exists for similar review types and the need for updating this literature is increasingly being acknowledged and addressed, for instance, in the PRISMA 2020 extensions for LSRs. The results of this scoping review will inform other authors, researchers, and decision-makers and show them what guidance literature is available or needs to be updated.

## Conclusion

From this scoping review, we can conclude that there is some important evidence for guidance on LSRs available. In terms of the numbers of identified sources including guidance, there is a high frequency of guidance papers on conducting and publishing a LSR. However, we identified less guidance on the reporting of a LSR and the least guidance on the quality appraisal of LSRs.

When considering our results from the scoping review, there is a particular need to develop and publish more guidance on how to adequately report in LSRs. An updated LSR-specific guidance document on reporting can be highly relevant for LSR authors, reviewers, editors, and other stakeholders involved in the LSR process. The scoping review results on reporting guidance have been used as a precursor and have been applied to inform and prepare a project on developing a PRISMA 2020 checklist extension for LSRs. The findings on the categories other than the reporting LSRs could be used by further author teams to re-evaluate and update existing guidance on SRs. Hence, we identified major evidence gaps for guidance on LSR appraisal. The AMSTAR 2 tool, which is currently used to assess the quality of SRs is not updated yet for the use of LSRs. This could be considered for further research, since there is an emerging need to develop an AMSTAR2 tool extension for novel methodological approaches to evidence syntheses, such as LSRs. Data can be made available upon author request. 

### Supplementary Information

**Additional file 1.** **Box 1.** Study search strategy.

**Additional file 2:** **Table S1.** Differences between protocol and scoping review. **Table S2.** Preferred Reporting Items for Systematic reviews and Meta-Analyses extension for Scoping Reviews (PRISMA-ScR) Checklist. **Table S3.** List of extracted study characteristics and extracted items of the categories.

## References

[1] Elliott JH, Turner T, Clavisi O, Thomas J, Higgins JPT, Mavergames C (2014). Living systematic reviews: an emerging opportunity to narrow the evidence-practice gap. PLoS Med.

[2] Elliott JH, Synnot A, Turner T, Simmonds M, Akl E, McDonald S, et al. Living systematic review 1: introduction - the why, what, when and how. J Clin Epidemiol. 2017;91:23–30. 10.1016/j.jclinepi.2017.08.010 28912002

[3] Cochrane. Guidance for the production and publication of Cochrane living systematic reviews: Cochrane Reviews in living mode. 2019.

[4] Page MJ, McKenzie JE, Bossuyt PM, Boutron I, Hoffmann TC, Mulrow CD (2021). The PRISMA 2020 statement: an updated guideline for reporting systematic reviews. BMJ (Clinical research ed).

[5] Shea BJ, Reeves BC, Wells G, Thuku M, Hamel C, Moran J (2017). AMSTAR 2: a critical appraisal tool for systematic reviews that include randomised or non-randomised studies of healthcare interventions, or both. BMJ.

[6] Peters M, Godfrey C, McInerney P, Munn Z, Tricco A, Khalil H. Chapter 11: scoping reviews (2020 version). In: In: Aromataris E MZE, editor. JBI Manual for Evidence Synthesis, JBI, 2020.

[7] Iannizzi C, Akl E, Kahale L, Dorando E, Mosunmola Aminat A, Barker J, et al. Methods and guidance on conducting, reporting, publishing and appraising living systematic reviews: a scoping review protocol. F1000Res. 2021;10:802. 10.12688/f1000research.55108.1 PMC8822136 35186269

[8] Arksey H, O'Malley L (2005). Scoping studies: towards a methodological framework. Int J Soc Res Methodol.

[9] Tricco AC, Lillie E, Zarin W, O'Brien KK, Colquhoun H, Levac D (2018). PRISMA extension for scoping reviews (PRISMA-ScR): checklist and explanation. Ann Intern Med.

[10] Khamis A, Kahale L, Pardo-Hernandez H, Schünemann H, Akl E. Methods of conduct and reporting of living systematic reviews: a protocol for a living methodological survey. F1000Res. 2019;8:221 10.12688/f1000research.18005.1 PMC6556985 31231512

[11] Kahale L, Elkhoury R, El Mikati I, Pardo-Hernandez H, Khamis A, Schünemann H, et al. Tailored PRISMA 2020 flow diagrams for living systematic reviews: a methodological survey and a proposal. F1000Res. 2021;10:192. 10.12688/f1000research.51723.1 PMC8804909 35136567

[12] Higgins JPT TJ, Chandler J, Cumpston M, Li T, Page MJ, Welch VA (editors). Cochrane Handbook for Systematic Reviews of Interventions version 6.2 (updated February 2021). Cochrane. 2021.

[13] Crequit P, Boutron I, Meerpohl J, Williams HC, Craig J, Ravaud P (2020). Future of evidence ecosystem series: 2. current opportunities and need for better tools and methods. J J Clin Epidemiol..

[14] Akl EA, Meerpohl JJ, Elliott J, Kahale LA, Schunemann HJ, Living Systematic Review N. Living systematic reviews: 4. Living guideline recommendations. J Clin Epidemiol. 2017;91:47–53. 10.1016/j.jclinepi.2017.08.009 28911999

[15] Harrington L. COVID-19 technology-enabled living systematic reviews to enhance knowledge translation. J AACN Adv Crit Care. 2021;32:133–6. 10.4037/aacnacc2021948 34161967

[16] Lansky A, Wethington HR (2020). Living systematic reviews and other approaches for updating evidence. J Am J Public Health.

[17] Lerner I, Crequit P, Ravaud P, Atal I (2019). Automatic screening using word embeddings achieved high sensitivity and workload reduction for updating living network meta-analyses. J J Clin Epidemiol.

[18] Macdonald H, Loder E, Abbasi K (2020). Living systematic reviews at the BMJ. J BMJ.

[19] Millard T, Synnot A, Elliott J, Green S, McDonald S, Turner T (2019). Feasibility and acceptability of living systematic reviews: results from a mixed-methods evaluation. J Syst Rev.

[20] Negrini S, Ceravolo MG, Cote P, Arienti C (2021). A systematic review that is “rapid” and “living”: a specific answer to the COVID-19 pandemic. J J Clin Epidemiol.

[21] Ravaud P, Crequit P, Williams HC, Meerpohl J, Craig JC, Boutron I (2020). Future of evidence ecosystem series: 3 From an evidence synthesis ecosystem to an evidence ecosystem. J J Clin Epidemiol..

[22] Simmonds M, Salanti G, McKenzie J, Elliott J, Living Systematic Review N (2017). Living systematic reviews: 3 Statistical methods for updating meta-analyses. J J Clin Epidemiol..

[23] Slaughter LA, Berntsen CF, Brandt L, Mavergames C. Enabling living systematic reviews and clinical guidelines through semantic technologies. J D-Lib Magazine. 2015;21.

[24] Ter Schure J, Grunwald P. Accumulation bias in meta-analysis: the need to consider time in error control. F1000Res. 2019;8:962 10.12688/f1000research.19375.1 PMC6808047 31737258

[25] Thomas J, Noel-Storr A, Marshall I, Wallace B, McDonald S, Mavergames C (2017). Living systematic reviews: 2. Combining human and machine effort. J J Clin Epidemiol..

[26] Thomas J, Askie L, Berlin J, Elliott J, Ghersi D, Simmonds M, et al. Chapter 22: Prospective approaches to accumulating evidence. In: Higgins JPT TJ, Chandler J, Cumpston M, Li T, Page MJ, Welch VA (editors), editor. Cochrane Handbook for Systematic Reviews of Interventions version 62 (updated February 2021): Cochrane; 2021.

[27] Vergara-Merino L, Verdejo C, Carrasco C, Vargas-Peirano M (2020). Living systematic review: new inputs and challenges. J Medwave.

[28] Winters M, de Vos RJ, van Middelkoop M, Rathleff MS, Weir A (2020). Stay alive! what are living systematic reviews and what are their advantages and challenges?. J Brit J Sports Med.

[29] Xu J, Deng H (2020). A brief introduction of living systematic review [Chinese]. J Chin J Evid-Based Med.

