# Supplementary material for: Methods and guidance on conducting, reporting, publishing, and appraising living systematic reviews: a scoping review
Source: Syst Rev. 2023 Dec 14;12:238. doi: 10.1186/s13643-023-02396-x (PMC10722674; doi:10.1186/s13643-023-02396-x)
Supplement: Supplementary file 1 — Additional file 1. Box 1. Study search strategy. [file 13643_2023_2396_MOESM1_ESM.docx]

Medline (via Ovid) and Epub Ahead of Print, In-Process, In-Data-Review & Other Non-Indexed Citations, Daily and Versions(R) 1946 to August 11, 2021 *(note on 26.08.2021 “evidence ecosystem” was added as keyword in the search strategy)*

# Searches

1 exp meta-analysis as topic/

2 Systematic Reviews as Topic/

3 (meta-analysis or review or systematic review).pt. or search*.tw.

4 ((continuous* or continual* or continue or periodic*) adj3 (updat* or search*)).mp.

5 (1 or 2 or 3) and 4

6 ((living adj3 (review* or metaanaly* or (meta adj analy*) or metanaly*)) or lsr).mp.

7 evidence ecosystem*.tw,kf.

8 5 or 6 or 7

9 limit 8 to yr="2013 -Current"

Cochrane Library (2021, Issue 08) via the Cochrane Library (searched 11 August 2021) *(note on 26.08.2021 “evidence ecosystem” was added as keyword in the search strategy)*

ID Search

#1 MeSH descriptor: [Meta-Analysis] explode all trees

#2 MeSH descriptor: [Meta-Analysis as Topic] explode all trees

#3 MeSH descriptor: [Systematic Review] explode all trees

#4 MeSH descriptor: [Systematic Reviews as Topic] explode all trees

#5 (meta-analysis or review or systematic review):pt

#6 search*:ti,ab

#7 ((continuous* OR continual* OR continue OR periodic*) NEAR/2 (updat* or search*)):ti,ab,kw

#8 (#1 OR #2 OR #3 OR #4 OR #5 OR #6) AND #7

#9 (living NEAR/2 (evidence OR metaanaly* OR meta-analy* OR metanaly*) OR LSR):ti,ab,kw

#10 evidence ecosystem*:ti,ab,kw

#11 #8 OR #9 OR #10 with Cochrane Library publication date Between Jan 2013 and Aug 2021

Embase (via Ovid) 1974 to August 11, 2021 *(note on 26.08.2021 “evidence ecosystem” was added as keyword in the search strategy)*

# Searches

1 exp meta analysis/ or "meta analysis (topic)"/

2 "systematic review"/ or "systematic review (topic)"/

3 (meta analys*s or review* or metanalys*s).ti. or search*.tw.

4 ((continuous* or continual* or continue or periodic*) adj3 (updat* or search*)).tw,kw.

5 (1 or 2 or 3) and 4

6 ((living adj3 (review* or metaanaly* or meta analy* or metanaly*)) or lsr).tw,kw.

7 evidence ecosystem*.tw,kw.

8 5 or 6 or 7

9 limit 8 to yr="2013 -Current"

10 limit 9 to medline

11 9 not 10

Box 1. Study search strategy
