# Supplementary material for: Methods and guidance on conducting, reporting, publishing, and appraising living systematic reviews: a scoping review
Source: Syst Rev. 2023 Dec 14;12:238. doi: 10.1186/s13643-023-02396-x (PMC10722674; doi:10.1186/s13643-023-02396-x)
Supplement: Supplementary file 2 — Additional file 2: Table S1. Differences between protocol and scoping review. Table S2. Preferred Reporting Items for Systematic reviews and Meta-Analyses extension for Scoping Reviews (PRISMA-ScR) Checklist. Table S3. List of extracted study characteristics and extracted items of the categories. [file 13643_2023_2396_MOESM2_ESM.docx]

|  | Protocol | Scoping review |
| --- | --- | --- |
| Search strategy | No clear date was given. We stated to search “from 2013”. | “We searched in MEDLINE, EMBASE, and Cochrane **on 28 August 2021** using the OVID interface.”  The search strategy was adjusted and slightly changed. Also, as the search strategy was slightly changes, we removed the restriction of searching only from 2013 on. |
| Data extraction | Extractors were not clarified. | Extractors were clarified. |
| Inclusion criteria | “We included articles that devoted at least **two** paragraphs to discuss methods or conceptual approaches for how to conduct, report, publish or appraise LSRs.” | “We included articles that devoted at least **one** paragraphs to discuss methods or conceptual approaches for how to conduct, report, publish or appraise LSRs.” |
| Exclusion criteria |  | Added: non LSR-specific papers |

Supplement table 1. Differences between protocol and scoping review

| **SECTION** | **ITEM** | **PRISMA-ScR CHECKLIST ITEM** | **REPORTED ON PAGE #** |
| --- | --- | --- | --- |
| **TITLE** | | | |
| Title | 1 | Identify the report as a scoping review. | 1 |
| **ABSTRACT** | | | |
| Structured summary | 2 | Provide a structured summary that includes (as applicable): background, objectives, eligibility criteria, sources of evidence, charting methods, results, and conclusions that relate to the review questions and objectives. | 2 |
| **INTRODUCTION** | | | |
| Rationale | 3 | Describe the rationale for the review in the context of what is already known. Explain why the review questions/objectives lend themselves to a scoping review approach. | 4 |
| Objectives | 4 | Provide an explicit statement of the questions and objectives being addressed with reference to their key elements (e.g., population or participants, concepts, and context) or other relevant key elements used to conceptualize the review questions and/or objectives. | 4 |
| **METHODS** | | | |
| Protocol and registration | 5 | Indicate whether a review protocol exists; state if and where it can be accessed (e.g., a Web address); and if available, provide registration information, including the registration number. | 5 |
| Eligibility criteria | 6 | Specify characteristics of the sources of evidence used as eligibility criteria (e.g., years considered, language, and publication status), and provide a rationale. | 5 |
| Information sources* | 7 | Describe all information sources in the search (e.g., databases with dates of coverage and contact with authors to identify additional sources), as well as the date the most recent search was executed. | 5 |
| Search | 8 | Present the full electronic search strategy for at least 1 database, including any limits used, such that it could be repeated. | 5 |
| Selection of sources of evidence† | 9 | State the process for selecting sources of evidence (i.e., screening and eligibility) included in the scoping review. | 6 |
| Data charting process‡ | 10 | Describe the methods of charting data from the included sources of evidence (e.g., calibrated forms or forms that have been tested by the team before their use, and whether data charting was done independently or in duplicate) and any processes for obtaining and confirming data from investigators. | 6 |
| Data items | 11 | List and define all variables for which data were sought and any assumptions and simplifications made. | Supplement table 2 |
| Critical appraisal of individual sources of evidence§ | 12 | If done, provide a rationale for conducting a critical appraisal of included sources of evidence; describe the methods used and how this information was used in any data synthesis (if appropriate). | / |
| Synthesis of results | 13 | Describe the methods of handling and summarizing the data that were charted. | 6 & 7 |
| **RESULTS** | | | |
| Selection of sources of evidence | 14 | Give numbers of sources of evidence screened, assessed for eligibility, and included in the review, with reasons for exclusions at each stage, ideally using a flow diagram. | 7 |
| Characteristics of sources of evidence | 15 | For each source of evidence, present characteristics for which data were charted and provide the citations. | 8 |
| Critical appraisal within sources of evidence | 16 | If done, present data on critical appraisal of included sources of evidence (see item 12). | / |
| Results of individual sources of evidence | 17 | For each included source of evidence, present the relevant data that were charted that relate to the review questions and objectives. | 9-15 |
| Synthesis of results | 18 | Summarize and/or present the charting results as they relate to the review questions and objectives. | 8 |
| **DISCUSSION** | | | |
| Summary of evidence | 19 | Summarize the main results (including an overview of concepts, themes, and types of evidence available), link to the review questions and objectives, and consider the relevance to key groups. | 15 |
| Limitations | 20 | Discuss the limitations of the scoping review process. | 15 |
| Conclusions | 21 | Provide a general interpretation of the results with respect to the review questions and objectives, as well as potential implications and/or next steps. | 15 |
| **FUNDING** | | | |
| Funding | 22 | Describe sources of funding for the included sources of evidence, as well as sources of funding for the scoping review. Describe the role of the funders of the scoping review. | 16 |
| JBI = Joanna Briggs Institute; PRISMA-ScR = Preferred Reporting Items for Systematic reviews and Meta-Analyses extension for Scoping Reviews.  * Where *sources of evidence* (see second footnote) are compiled from, such as bibliographic databases, social media platforms, and Web sites.  † A more inclusive/heterogeneous term used to account for the different types of evidence or data sources (e.g., quantitative and/or qualitative research, expert opinion, and policy documents) that may be eligible in a scoping review as opposed to only studies. This is not to be confused with *information sources* (see first footnote).  ‡ The frameworks by Arksey and O’Malley (6) and Levac and colleagues (7) and the JBI guidance (4, 5) refer to the process of data extraction in a scoping review as data charting*.*  § The process of systematically examining research evidence to assess its validity, results, and relevance before using it to inform a decision. This term is used for items 12 and 19 instead of "risk of bias" (which is more applicable to systematic reviews of interventions) to include and acknowledge the various sources of evidence that may be used in a scoping review (e.g., quantitative and/or qualitative research, expert opinion, and policy document). | | | |

Supplement table 2. Preferred Reporting Items for Systematic reviews and Meta-Analyses extension for Scoping Reviews (PRISMA-ScR) Checklist

| Extracted data | Detailed sub-items extracted |
| --- | --- |
| Study characteristics | - Main objective - Type of publication - Methods - Journal - Type of LSR guidance (conducting, reporting, publishing, appraisal of LSRs) |
| Items for guidance on conducting LSR | - Criteria/rationale for conducting LSR - Evidence on inclusion criteria - Evidence on search - Evidence on data extraction - Evidence on quality & bias assessment - Evidence on data synthesis with meta-analysis (if applicable) - Evidence on certainty of the evidence assessment - Authorship changes - Ongoing method support - Funding |
| Items for guidance on reporting LSR | 27 items from the original PRISMA statement checklist |
| Items for guidance on publishing LSR | - Publication type of new findings - Publication of review status - New citation & added to PubMed - Publication of an update - Publication of between updates information - Transition out of living mode - Peer review updates - Publish authorship changes - Publication of Prisma flow diagram |
| Items for guidance on appraising LSR | - (1)RQ & inclusion criteria - (2)Methods established prior to the conduct & justify deviation from protocol - (3)Explain study selection - (4)Use of comprehensive search strategy - (5)Study selection in duplicate - (6)Data extraction in duplicate - (7)List of excluded studies & justification - (8)Adequate description of included studies - (9)Use of appropriate RoB assessment technique - (10)Funding of included studies reported - (11)Use of appropriate methods for meta-analysis - (12)(if meta-analysis) assessment of potential RoB impact on pooled results - (13)Accounted for RoB when interpreting/discussing the results - (14)Explanation & discussion of heterogeneity observed in results - (15)(if quantitative synthesis) adequate investigation of publication bias & impact on result - (16)Report of potential COI sources (funding) - Use & handling of preprints - Guidance on using a specific checklist |

Supplement table 3. List of extracted study characteristics and extracted items of the categories
